# The Y chromosome: a blueprint for men’s health?

**DOI:** 10.1038/ejhg.2017.128

**Published:** 2017-08-30

**Authors:** Akhlaq A Maan, James Eales, Artur Akbarov, Joshua Rowland, Xiaoguang Xu, Mark A Jobling, Fadi J Charchar, Maciej Tomaszewski

**Affiliations:** 1 Division of Cardiovascular Sciences, Faculty of Biology, Medicine and Health, University of Manchester, Manchester, UK; 2 Department of Genetics, University of Leicester, Leicester, UK; 3 School of Applied and Biomedical Sciences, Faculty of Science and Technology, Federation University, Mount Helen Campus, Ballarat, VIC, Australia; 4 Division of Medicine, Central Manchester NHS Foundation Trust, Manchester Academic Health Science Centre, Manchester, UK

## Abstract

The Y chromosome has long been considered a ‘genetic wasteland’ on a trajectory to completely disappear from the human genome. The perception of its physiological function was restricted to sex determination and spermatogenesis. These views have been challenged in recent times with the identification of multiple ubiquitously expressed Y-chromosome genes and the discovery of several unexpected associations between the Y chromosome, immune system and complex polygenic traits. The collected evidence suggests that the Y chromosome influences immune and inflammatory responses in men, translating into genetically programmed susceptibility to diseases with a strong immune component. Phylogenetic studies reveal that carriers of a common European lineage of the Y chromosome (haplogroup I) possess increased risk of coronary artery disease. This occurs amidst upregulation of inflammation and suppression of adaptive immunity in this Y lineage, as well as inferior outcomes in human immunodeficiency virus infection. From structural analysis and experimental data, the *UTY* (*Ubiquitously Transcribed Tetratricopeptide Repeat Containing, Y-Linked*) gene is emerging as a promising candidate underlying the associations between Y-chromosome variants and the immunity-driven susceptibility to complex disease. This review synthesises the recent structural, experimental and clinical insights into the human Y chromosome in the context of men’s susceptibility to disease (with a particular emphasis on cardiovascular disease) and provides an overview of the paradigm shift in the perception of the Y chromosome.

## A shift in thinking

Views on the biological importance of the Y chromosome have peaked and troughed over the past 60 years.^1, 2, 3^ Although its perception as the key determinant of male sex has remained fundamentally unchanged, the potential association between the Y and human disease has been much more ambiguous.

In the second half of the twentieth century, there was much interest in holding this unique chromosome to account for so-called ‘Y-linked’ or ‘holandric’ traits.^4^ At least 14 such traits generated interest because of their exclusive father-to-son transmission, including hypertrichosis pinnae auris (HPA) – having abnormally long hair on the outer ear (pinna). Despite problems of reporter bias and illegitimacy in the numerous pedigrees studied,^1, 5, 6^ the pattern of inheritance suggested that HPA was a Y-linked trait. This was until a 2004 study^7^ utilised contemporary Y-chromosome haplogroup classification to show that no single haplogroup predominated in the HPA cases, thus making it unlikely to be Y linked and more likely to be an autosomal trait with phenotypic expression limited to males.

Several small case–control studies conducted in the 1970s found associations between the 47,XYY karyotype (males carrying two copies of the Y chromosome) and criminality^8^ and between the length of Y and physical activity levels.^9^ These studies exemplify the simmering interest at the time in linking the Y chromosome to psychological/physiological phenotypes. However, doubts about the validity of these Y-linkage studies^2^ led to an era dominated by the idea that the Y chromosome possessed little genetic content, and that its sole purpose was to trigger testis development in males.^10^ Such was the redundancy assigned to the Y chromosome that some commentators described it as a ‘genetic wasteland’ during this period.^3^

Subsequent work on the Y chromosome during the 1990s reinforced its role in the reproductive system with the localisation of the specific testis-determining factor to *sex-determining region Y (SRY)*^11^ and the definition of three distinct loci involved in spermatogenesis (*azoospermia factor a*, *b* and *c*), deletions of which are associated with varying degrees of spermatogenic failure in men.^12^

Recent advances in genetic technologies enabling mapping and sequencing of the Y chromosome have again altered scientific perspectives on the Y. Although the Human Genome Project confirmed that there is relatively sparse protein-coding material within the Y and a high degree of repetition, the finding that more than half of the active genes are expressed in non-gonadal tissue throughout the body^3^ has brought the Y to the forefront of research on men’s susceptibility to disease.

## Structure of the Y chromosome

Comprehensive sequencing of the Y chromosome was first completed in 2003 by Skaletsky *et al*^3^ and their findings remain largely valid today.

The male-specific region of the Y chromosome (MSY) makes up 95% of its length.^3^ Unlike autosomal chromosomes, the MSY does not undergo reciprocal recombination with a partner chromosome during meiosis.^3^ Only short regions at either tip of the chromosome undergo recombination with the X, and these are called the pseudoautosomal regions (PARs).^3^ Because of the lack of crossing over, the MSY is transmitted unaltered from father to son along the paternal line.^3^ Using a combination of genetic markers (usually single-nucleotide polymorphisms (SNPs)) it is possible to classify each individual Y chromosome into one of numerous haplogroups.^13^ In 2002, the Y Chromosome Consortium collated all phylogenetically informative SNPs discovered to date, constructing a robust maximum-parsimony tree, and assigning universal nomenclature to each recognised haplogroup;^14^ subsequently, this was updated through sporadic SNP discovery,^15^ and more recently thanks to large-scale resequencing projects that have yielded tens of thousands of SNPs.^16, 17, 18^ To simplify the task of selecting suitable SNPs for genotyping in medical and other studies, a stable minimal phylogeny containing 417 SNPs has also been described.^19^ The phylogenetic framework has been incorporated into studies of association between the Y chromosome and susceptibility to complex diseases. This important development has filled (at least to some extent) a void in genetic association discovery for the Y chromosome left by genome-wide association studies (GWASs) in which, due to its haploid nature, the Y chromosome was routinely ignored.^20^

**Figure 1:**
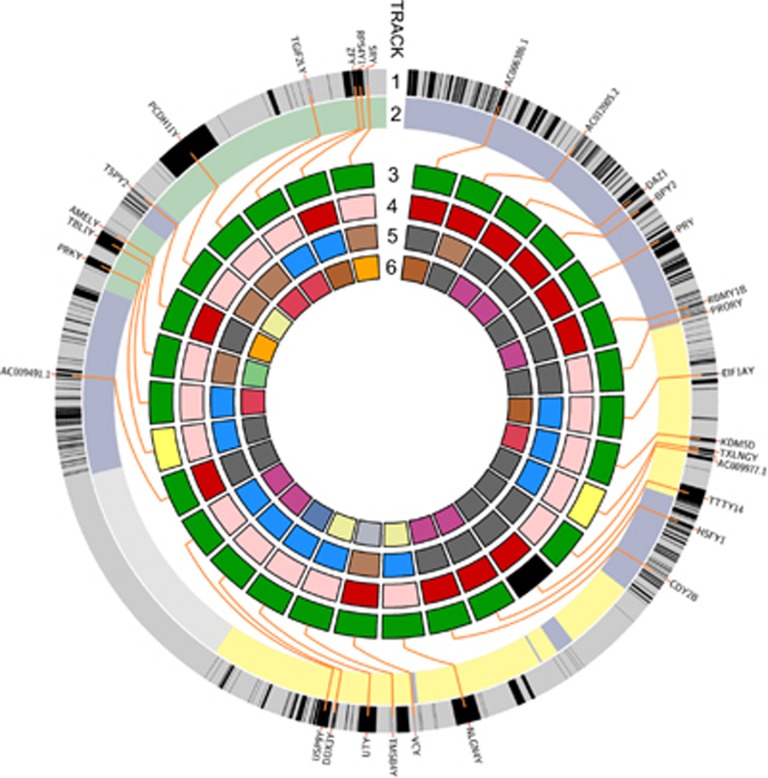
Genes of the Y chromosome. Chromosome starts at the top of the circle and proceeds anticlockwise. Track 1=locations and lengths of Y genes: Ensembl v86 genes are shown as black tiles, with genes that either encode distinct MSY proteins or are known to produce biologically significant products labelled. Please note that the protein-coding gene *AC009977.1* lies within the Y-chromosome coordinates for *TXLNGY* but is positioned on the reverse strand rather than the forward strand. For greater visual identification and separation of tiles for these two genes, the position of *AC009977.1* has been shifted slightly proximally. Track 2=Y-chromosome regions: this track represents gross structural subdivisions of the Y. Green=short arm (Yp); light purple=ampliconic regions; grey=centromere; yellow=long arm (Yq). Track 3=Gene biotype: this track illustrates the current Ensembl biotype status for each of the labelled Y genes. Green=protein-coding; yellow=pseudogene; black=noncoding RNA. Track 4=Copy/isoform number: this track represents the number of copies or isoforms that each gene possesses on the Y chromosome. Single copy=light pink; multicopy=solid, dark red. Track 5=X paralogue and/or X–Y gene dosage sensitive: this track shows genes that have an X paralogue and/or have been classified as one of 12 X–Y dosage-sensitive gene pairs. Dark grey=gene has neither X paralogue nor is part of an X–Y gene dosage-sensitive pair; brown=gene has an X paralogue but is not part of an X-Y gene dosage-sensitive pair; blue=gene possesses an X paralogue and is part of an X–Y gene dosage-sensitive pair. Track 6=Biological functions: known or potential biological functions of the gene products. Brown=translation, red=transcription, pink=spermatogenesis, light yellow=cell adhesion, light green=biomineralisation, blue=T-cell activation, dark grey=unknown, light grey=brain development, orange=cell differentiation. Plot constructed using Circos software.^66^

Compared with all other nuclear chromosomes, the Y harbours the smallest number of genes at 568 and is considerably shorter than the X chromosome in length (~57 Mbp compared with ~156 Mbp).^21^ Based on Ensembl data (v86), only 71 of the Y-chromosome genes have protein-coding potential; however, several of the encoded proteins belong to the same protein families, leaving only 27 genes coding for distinct MSY proteins.^21^ These 27 protein-coding genes are displayed in Figure 1. The 109 genes produce long and short noncoding RNAs, all of which could have important effects on regulation of gene expression, but none of which have had their regulatory potential explored in greater detail.^21^ The remaining 388 genes are described as pseudogenes.^21^ It is important to note that many of the gene assignments are predictions and require biological validation. Figure 1 highlights key structural and functional characteristics of the 27 Y-chromosome genes that encode distinct MSY proteins and three noncoding genes. For full names of genes mentioned in the main text and figures, please see Table 1.

**Figure 2:**
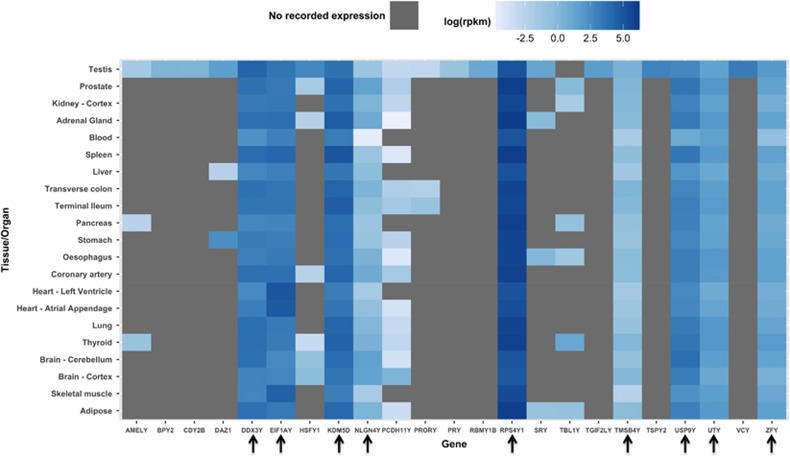
Tissue expression of key Y-chromosome genes. This heatmap illustrates the relative tissue expression in a range of different tissues for 23 out of 27 Y-chromosome genes that encode distinct MSY proteins. The tissue expression profiles for protein-coding genes *AC006386.1*, *AC009491.1*, *AC009977.1* and *AC012005.2* have not been evaluated and validated at the time of this review and hence these genes have not been included in the heatmap. Data are based on RNA transcript values for each gene (Reads Per Kilobase of transcript per Million mapped reads (RPKM)) obtained from GTex Portal^22^ that have been transformed logarithmically. Lighter shades of blue represent lower log(RPKM) values and lower levels of expression in the particular tissue, whereas darker shades of blue represent higher log(RPKM) values and higher levels of expression in the particular tissue. Grey blocks represent no recorded expression of the gene in the tissue of interest. The nine genes that are ubiquitously expressed have been labelled with an arrow below the gene name.

Of the 27 genes that encode distinct MSY protein, 9 are ubiquitously expressed; a further 14 are considered testis specific or show predominant expression in specific tissues such as the brain (eg, *PCDH11Y*) or the thyroid (eg, *TBL1Y*);^22^ the remaining 4 do not currently have validated tissue expression data available – Figure 2 shows the pattern of tissue expression for the 23 protein-coding genes whose tissue expression has been evaluated and validated. Many of the original genes described as testis-specific by Skaletsky *et al*^3^ have now been reclassified as ubiquitous using updated tissue expression data.^22^ All of the latter are X-degenerate genes – they have a paralogue on the X chromosome.^3^

### X–Y gene dosage

Recent work by Bellott *et al*^23^ has identified 12 X–Y gene pairs that are collectively critical for survival (see track 5 in Figure 1). The X paralogue of each pair escapes X inactivation, implying a dose-sensitive relationship that requires both genes to be active.^23^ These genes are generally ubiquitously expressed and are understood to perform a variety of gene expression regulatory functions including chromatin modification, splicing and translation,^23^ and are thus potentially relevant to a wide range of physiological traits and susceptibility to disease.

## Hypertension

Genetic crosses of spontaneously hypertensive rats (SHRs) and normotensive Wistar Kyoto (WKY) rats produced male offspring of SHR fathers with significantly higher blood pressure compared with the offspring of SHR mothers.^24^ Consomic techniques have been used to isolate and estimate approximate contributions of the Y chromosome and autosomes to blood pressure.^25^ Here, successive selective breeding of male offspring across several generations leads to male rats that possess the Y chromosome of interest on a known, defined genetic background of autosomes and X chromosome from the normal strain.^25^ This allows for isolated analysis of the phenotypic effects of the Y.^25^ To study Y-chromosome influence in the SHR phenotype, Turner *et al*^25^ developed two separate consomic strains: one strain possessing the Y chromosome from the SHR rat, X and autosomal chromosomes from the normotensive WKY rat and another with the opposite configuration.^25^ Such techniques revealed that the Y chromosome independently raised blood pressure by 34 mm Hg.^25^ A later review by Ely *et al*^26^ estimated the Y effect on blood pressure at a more modest 15–20 mm Hg. A linkage of Y to blood pressure has also been demonstrated in rat strains other than the Wistar Kyoto.^27^

Search for a potential locus mediating this effect in rats has focused on the *SRY* gene, already well established as the testis-determining factor. Whereas humans possess a single copy of *SRY* on the Y, normotensive experimental rats carry multiple highly similar copies.^28^ Sequencing techniques show the presence of an additional *SRY3* copy in the SHR,^29, 30^ containing a proline-to-threonine amino acid substitution at position 76.^30^ Importantly, SRY is a transcription factor that, in synergy with androgen receptor and in a testosterone-dependent manner,^30^ regulates promoter regions for genes encoding angiotensinogen, renin, angiotensin-converting enzyme (ACE) and ACE2^29^ – known for their key roles in blood pressure regulation. The threonine point mutation in *SRY3* has been shown to reduce SRY3 promoter regulation,^30^ leading to an increase in transcription of angiotensinogen, renin and ACE, thus promoting formation of the vasoconstrictor angiotensin II (Ang II); in contrast, *SRY3* has an inhibitory effect on ACE2 transcription, the enzyme important for formation of vasodilatory and blood pressure-lowering Ang-(1–7).^29^ Experimental delivery of *SRY3* to normotensive rat kidneys raises blood pressure,^30, 31^ a rise that can be prevented by concomitant administration of olmesartan, a renin–angiotensin–aldosterone system (RAAS) inhibitor.^30^ The *in silico* analysis and transfection studies of Chinese Hamster Ovary cells show that the *SRY* X paralogue (*SOX3*) is also capable of influencing RAAS gene expression, although *in vivo SOX3* is primarily transcribed in non-kidney tissues.^32^ This suggests that the *SRY* paralogue is unique in its pro-hypertensive effects in male rats.

The translatability of *SRY* as a key blood pressure regulator to humans is uncertain. Rat *SRY* is significantly different to that of humans not only in terms of copy number but also in terms of the gross protein structure: human *SRY* lacks a polyglutamine (Q-)-rich motif present in rat *SRY* and the high-mobility group (HMG)-box region important for DNA binding is in a different location.^28^ Nevertheless, human *SRY* has been shown to influence expression of rat and human RAAS genes *in vitro*,^33^ suggesting potential to play a role in genetically acquired human hypertension.

One of the earliest studies to suggest that blood pressure could be a Y-influenced phenotype in humans evaluated Japanese university students aged 17–21 years with and without hypertensive parents.^34^ Male students born to hypertensive fathers had significantly higher systolic and diastolic blood pressures than female students born to hypertensive fathers, suggesting a possible genetic susceptibility to higher blood pressure via paternal lineage and/or autosomal influence that was sex limited.^34^ However, the absence of significant difference in blood pressure between male students born to hypertensive mothers or fathers^34^ seemed to argue against Y linkage. Although these results were inconclusive, they prompted a series of studies to investigate associations between specific genetic variants of the Y chromosome and hypertension.

One extensively studied variant is a *Hin*dIII restriction site polymorphism in the Y-chromosomal alphoid satellite DNA that divides Y chromosomes into two classes.^35^ The majority of these studies preceded the introduction of informative phylogenetic tree classification; however, the class showing absence of the restriction site is equivalent to the currently defined super-haplogroup P-M45.^36^ Whereas some studies found an association between *Hin*dIII variants and altered systolic/diastolic blood pressure with effect sizes ranging from 1.44 to 6.2 mm Hg,^35, 37, 38^ others failed to replicate this association.^39, 40, 41, 42, 43^

Phylogenetically based studies using haplogrouping strategies are considered a more efficient method of identifying an association between the Y chromosome and a phenotype compared with single, isolated variants. Such studies by our group^44, 45^ found no evidence of association between one of the most common European lineages of the Y chromosome (haplogroup I) and blood pressure. However, this does not rule out associations between other common haplogroups and blood pressure. Delineating such associations will benefit greatly from extensive databases such as the UK Biobank that includes phenotypic data for 230 000 men, genotyped using an array of markers with extensive coverage for the Y chromosome, thus facilitating haplogrouping.^46^

The evidence currently points to a strong Y-chromosome signal influencing blood pressure in rats with a relative paucity of evidence in human studies. The human genetic association studies had inherent limitations related to the availability of genotyping technology when they were conducted (only one to two polymorphisms were studied at a time). Currently, there are no convincing data that even if a blood pressure regulating gene exists on the human Y, it is the same gene that stimulates the blood pressure rise in rodents.

## Coronary artery disease

Our earlier study established an association between the Y chromosome and coronary artery disease (CAD) in two separate British cohorts using reconstruction of the Y phylogeny.^44^ One of the most common European lineages, haplogroup I, was associated with a higher incidence of CAD compared with all others. This effect was present in both the cross-sectional British Heart Foundation Family Heart Study (BHF-FHS) and the prospective West of Scotland Coronary Prevention Study (WOSCOPS). Indeed, the magnitude of the effect was comparable across both studies, with the odds ratio for CAD with haplogroup I being 1.75 (95% CI 1.20–2.54, *P*=0.004) in BHF-FHS and 1.45 (95% CI 1.08–1.95, *P*=0.012) in WOSCOPS. In the prospective WOSCOPS, cardiovascular risk parameters were also available and haplogroup I was not associated with any traditional cardiovascular risk factors including hypertension, dyslipidaemia, high BMI, diabetes, elevated C-reactive protein (CRP), alcohol consumption or smoking. Importantly, the associations between haplogroup I and CAD were not affected by the adjustment for common autosomal variants linked to CAD identified in previous GWAS.

In search of molecular mechanisms that may explain the association between haplogroup I and CAD, we explored monocyte and macrophage transcriptomes of men whose Y chromosomes were genetically characterised and haplogrouped.^44^ This transcriptome-wide analysis revealed differences in expression of 30 Kyoto Encyclopedia of Genes and Genomes (KEGG) pathways between men with haplogroup I and carriers of other haplogroups. Nineteen of these pathways belonged to inflammatory or immune signalling cascades. In general, there was a downregulation of genes in pathways involved in autoimmunity and adaptive immunity (such as antigen processing and presentation) combined with upregulation of genes in inflammatory pathways (such as transendothelial leukocyte migration). This is highly pertinent given the significant inflammatory component to atherosclerosis,^47^ exhibited by monocyte entry into the intimal layer of arteries and subsequent differentiation into macrophages that internalise lipids and stimulate intimal hyperplasia.^48^

The male-related phenotypes such as aggression and sex steroid levels (including androstenedione, testosterone) showed no differences between men who inherited haplogroup I from their fathers and those representing other paternal lineages.^49^ As such, these factors most intuitively linked to male sex are unlikely to explain the association between haplogroup I and increased susceptibility to CAD.

Recent studies have looked at the role of Y haplogroup in determining risk of other cardiovascular diseases. A recent prospective study conducted in Cypriot men (in whom prevalence of haplogroup I is estimated at 2.4%) associated haplogroup K with a more than twofold increased risk of atherosclerotic plaque occurrence in the carotid and femoral artery bifurcations compared with all other haplogroups.^50^ Systolic blood pressure was also associated with haplogroup K in this analysis and was proposed as a potential intermediate phenotype of the identified association.^50^ In contrast, Haitjema *et al*^51^ studied histological vessel wall characteristics of Dutch patients who had undergone carotid endarterectomy or open aneurysmal repair, but found no significant differences in vessel wall characteristics including leukocyte infiltration, lipid, collagen and smooth muscle content between the major haplogroups present (including I with prevalence 24–28%). Caution should be exercised when interpreting the data from this analysis with the results of the previous study on CAD given the obvious differences between the mechanisms of CAD, carotid artery disease and abdominal aortic aneurysms.^51^

The association between haplogroup I and CAD presents a strong case for a Y-linked heritable component of CAD. The effects of haplogroup I are independent of traditional cardiovascular risk factors or the male-related phenotypes. It is anticipated that utilisation of larger cohorts (such as those derived from the UK Biobank) will increase the power to detect phenotypes that may mediate the link between haplogroup I and CAD.

## Immunity and inflammation

The immune system and inflammation play key roles in atherosclerosis and the ensuing development of CAD. In this context, the emerging evidence for the role of the Y-chromosome genes in immunity and the inflammatory response strengthens the hypothesis that the association between Y-chromosome haplogroup and CAD is mediated by the immune system.

### Viral infections

In a population of European Americans, CAD-predisposing haplogroup I was associated with faster progression of HIV to AIDS, a greater depletion of the CD4+ T-cell count and a higher mortality rate than other haplogroups more than 7 years after initial infection.^52^ Haplogroup I was also associated with a higher risk of malignancy, including the highly specific AIDS-defining malignancy Kaposi’s sarcoma.^52^ Moreover, individuals with haplogroup I were more resistant to highly active antiretroviral therapy, taking longer to achieve viral load suppression.^52^ This implies a prominent role for the Y chromosome in determining outcomes of HIV infection where systematic immune system targeting of virally infected CD4+ T cells is a key process underlying pathogenesis.^53^ Furthermore, *in vitro* studies show that *DDX3X* (the X paralogue of the Y gene *DDX3Y*) is a determinant of HIV-1 replication.^54^ These examples offer strong support for inherited differences in immune responses between men with haplogroup I and those from others.^44^

### UTY

Following the discovery of an association between haplogroup I and CAD, our group carried out gene expression analysis to compare the macrophage expression of X-degenerate genes of the MSY between men with CAD-predisposing haplogroup I and other haplogroups.^45^ Of the 14 X-degenerate genes with confirmed macrophage expression, 2 were associated with haplogroup I – men with this paternal lineage showed ∼0.61- and 0.64-fold lower expression of *UTY* (*Ubiquitously Transcribed Tetratricopeptide Repeat Containing, Y-Linked*) and *PRKY* (*Protein Kinase, Y-Linked*), respectively.^45^ Little is known about the biological functions of *PRKY*, although it is speculated to encode a ubiquitously expressed protein kinase that may have important signalling functions.^21^ The downregulation of *UTY*, on the other hand, is particular intriguing given the links of *UTY* and its X paralogue, *UTX*, with various aspects of inflammation and immunity.

**Figure 3:**
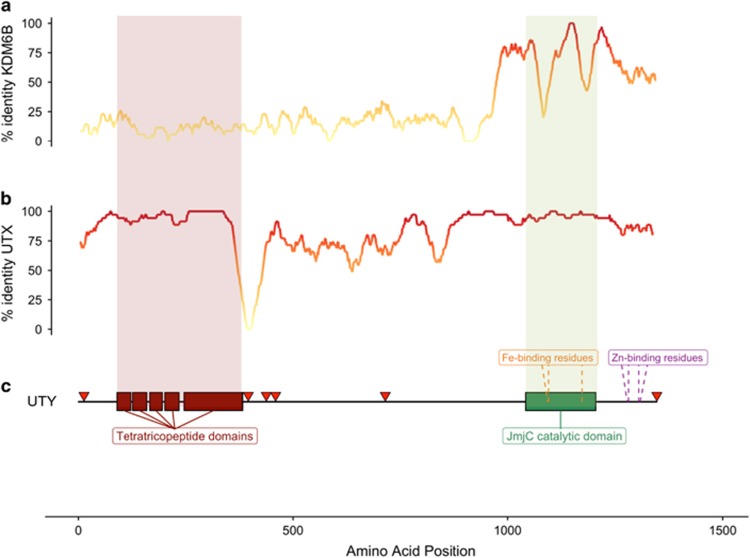
Amino acid sequence homology of UTY and related histone demethylases. (**a**) Line graph representing sequence identity of UTY aligned with KDM6B; the colour of the line correlates with the degree of sequence identity (yellow→orange→red colours show increasing % identity). (**b**) Line graph representing sequence identity of UTY aligned with UTX, and (**c**) the structure of the UTY protein including relative positions of the Tetratricopeptide domains and the JmjC catalytic domains, together with important iron (Fe) and zinc (Zn) ion binding residues. Red triangles represent deletions within UTY compared with UTX protein sequence. UTY possesses particularly high sequence identity with UTX in the Tetratricopeptide repeat domains and the JmjC catalytic domain as compared with other areas of the protein. Although UTY does not display conservation of the Tetratricopeptide domains of KDM6B, there is strikingly high conservation of the JmjC domain. These protein sequence similarities in important domains imply the possibility of UTY possessing functional histone demethylase activity. Protein sequence and domain data were obtained from UniProt.^61^ UniProt accession numbers of sequences used: O14607 (UTY), 015550 (UTX/KDM6A) and 015054 (KDM6B).

*UTY* encodes a minor histocompatibility antigen important for male stem cell allograft rejection^55^ – a process linked to one of the KEGG pathways associated with haplogroup I in transcriptome-wide analysis.^44, 56^ *UTX* is implicated in the proinflammatory response of macrophages.^57^ Structural analysis has identified a specific enzyme inhibitor of *UTX* and subsequent selective inhibition leads to a reduction in inflammatory cytokine release, including TNF-*α*, by human macrophages.^57^ Moreover, *in vitro* studies exemplify the importance of *UTX* for facilitation of T-follicular helper cell differentiation and indirectly the maturation of IgG-secreting plasma cells in the setting of chronic viral infections.^58^ *UTX* (also known as *KDM6A*) encodes an enzyme belonging to a family of lysine-specific histone demethylases (KDMs) that remove epigenetic marks at histone H3 Lysine 27 (H3K27).^59^ These KDMs regulate transcription and possess a Jumonji C (JmjC) domain that utilises iron as a cofactor.^60^ Protein sequence analysis suggests that *UTY* possesses particularly high sequence identity (≈96%) with *UTX* in two important domains: the tetratricopeptide repeat (TPR) regions and the JmjC catalytic domain that includes the principal iron-binding residues (Figure 3).^61^ *UTY* comparison with the autosomal-encoded, functional histone demethylase *KDM6B* (also known as *JMJD3*) suggests relatively high sequence homology in the pertinent JmjC domain but less so in the TPR regions (Figure 3) that have undetermined function.^60^ A high degree of conservation in the important JmjC domain region of *UTY* with functional KDMs suggests that *UTY* could be an active histone demethylase and, thus, implicated in similar inflammatory and immune processes to those associated with *UTX*. Indeed, although protein sequence analysis would suggest the high degree of JmjC sequence similarity between *UTY* and the functional KDMs (Figure 3), there is some conflict in the literature regarding whether *UTY* inherently possesses histone demethylase activity. *UTX* knockout experiments in mice embryos suggest that *UTY* exhibits redundancy for *UTX* activity.^59^ Follow-up *in vivo* analysis, however, found that both mouse and human *UTY* lacked inherent histone demethylase activity, suggesting that biological functions of UTY (and UTX) may be at least partly independent of the demethylase region.^59^ In contrast, a different group conducted *in vitro* analysis revealing conserved *UTY* histone demethylase activity, although reduced compared with *UTX*.^60^ Based on studies conducted to date, *UTY* appears capable of regulating gene expression, possibly (at least in part) via histone demethylation. It is tempting to speculate that altered *UTY* expression in carriers of haplogroup I may contribute to the observed changes in their macrophage expression of inflammatory and immune pathways. This will require further studies, in particular given the evidence for another Y gene (*KDM5D*, formerly known as *SMCY*) to exhibit histone H3 Lysine 4 (H3K4) demethylase activity^21^ and play a role in immunological complications of stem cell transplantation.^62^

### Autoimmunity

Haplogroup I was also associated with downregulation of pathways involved in human autoimmunity.^44^ Further support for the Y chromosome as a potential autoimmunity locus comes from animal models of diseases with significant autoimmune components such as experimental allergic encephalomyelitis (EAE) and experimental myocarditis.^63^ Indeed, experiments on consomic strains of mice showed that the Y chromosome defining the strain strongly influenced the susceptibility to and severity of EAE and myocarditis.^63^ Copy numbers of mouse Y genes, *SLY* and *RBMY*, were correlated with disease severity and the strains with reduced susceptibility carried fewer copies of these genes.^63^ In addition, transcriptomic analysis showed 398 differentially expressed Y-chromosome transcripts in the macrophages and CD4+ T cells between the more and less susceptible strains.^63^ These observations have been mirrored (at least to some extent) in male patients with an early form of multiple sclerosis – clinically isolated syndrome (CIS) – a disease with a strong autoimmune component and the human correlate of EAE.^63^ Compared with healthy controls, CD4+ T cells from individuals with CIS showed differential expression of a large proportion of the same Y genes identified in the mouse autoimmune models, suggesting a common Y-determined genetic basis to autoimmunity in mice and humans.^63^

## Future studies

With the emerging availability of large data sets comprising clinical phenotypes and Y-chromosome genotypes, future research should fully utilise the power of phylogenetic analysis to explore the potential contribution of Y chromosome to complex polygenetic traits. In particular, the evidence suggests the Y chromosome can be a powerful determinant of male immunity, including autoimmunity. We therefore propose studies to determine associations between Y-chromosome haplogroup and autoimmune disorders (such as rheumatoid arthritis), many of which exhibit sexual dimorphisms.^64^

In addition, trans-ethnic mapping studies would benefit our understanding of the complex relationship between Y-chromosome haplogroup and CAD. In the original British cohorts, two haplogroups predominated – R1b1b2 (70.0–72.7%) and I (14.5–17.0%).^44^ Although the elevated CAD risk associated with haplogroup I was attributed to haplogroup I posing increased susceptibility, an alternative interpretation of the results could be that the main non-I haplogroup (R1b1b2) offered protection against CAD. The uncertainty regarding precise identification of the causal haplogroup is exacerbated by two haplogroups accounting for nearly 90% of the cohort. Analyses of populations of different ethnicities (such as East Asians) with greater haplogroup diversity and an absence of haplogroup I^13^ would enable greater understanding of the specific cause for the altered CAD risk. If an association was found between haplogroup R (the clade containing R1b1b2 as a subtype) and reduced CAD risk in such a population, this would suggest that rather than haplogroup I elevating CAD risk, the causal factor for the original association was haplogroup R1b1b2-mediated protection against CAD. In contrast, an association between a haplogroup completely absent in Europe (eg, O) and CAD in East Asians would imply a greater range of unique genetic variants underpinning Y-mediated CAD risk. By analysing the locations of these variants, particular Y genes likely to have altered expression (variants within promoter sites) or altered molecular function of encoded proteins (variants within exon sequences) could be identified.

Further investigation of the inflammatory and immune systems as potential mediators of the link between Y lineage and CAD requires high-fidelity immune phenotyping studies. This should involve extensive RNA sequencing of a wide range of target immune cells and tissues involved in CAD pathogenesis (such as dendritic cells, T and B lymphocytes) to provide much needed insight into the expression of all MSY protein-coding genes and their influence on underlying inflammatory and immune responses. Furthermore, the functional roles of Y-chromosome long noncoding RNAs and pseudogenes, especially within control of genetic regulation, remain largely unexplored.^65^ It is anticipated that examination of Y non-protein-coding elements within the broader ENCODE project will identify potential functional pathways mediating the associations between the Y and disease.

## Conclusions

Data from association studies have revealed a potential role for the genetic variation within the Y chromosome in determination of men’s health and susceptibility to disease. This contrasts with initial pessimistic views about the Y as a futile, redundant piece of DNA. One of the strongest pieces of evidence is the association between haplogroup I and increased CAD risk, in the context of inflammation and immunity. Future endeavours will need to concentrate on identifying specific MSY genes that directly influence inflammatory and adaptive immunity processes within atherosclerosis. The identification of 12 X–Y dosage-sensitive gene pairs has refined our focus for future studies. Three of these pairs warrant further attention given their prior associations with haplogroup I and/or immune processes: *UTY/UTX*, *PRKY/PRKX* and *KDM5D/KDM5C*, with UTY being the most promising functional candidate.

## Figures and Tables

**Table 1:** Gene abbreviations and acronyms used in text

*AC006386.1*	No full gene name
*AC009491.1*	No full gene name
*AC009977.1*	No full gene name
*AC012005.2*	No full gene name
*AMELY*	*Amelogenin, Y-Linked*
*BPY2*	*Basic Charge, Y-Linked, 2*
*CDY2B*	*Chromodomain Y-Linked 2B*
*DAZ1*	*Deleted In Azoospermia 1*
*DDX3X*	*DEAD-Box Helicase 3, X-Linked*
*DDX3Y*	*DEAD-Box Helicase 3, Y-Linked*
*EIF1AY*	*Eukaryotic Translation Initiation Factor 1A, Y-Linked*
*HSFY1*	*Heat Shock Transcription Factor, Y-Linked 1*
*JMJD3*	*Jumonji Domain-Containing 3*
*KDM5C*	*Lysine-specific Demethylase 5C*
*KDM5D*	*Lysine-specific Demethylase 5D*
*KDM6A*	*Lysine Demethylase 6A*
*KDM6B*	*Lysine-specific Demethylase 6B*
*NLGN4Y*	*Neuroligin 4, Y-Linked*
*PCDH11Y*	*Protocadherin 11 Y-linked*
*PRKX*	*Protein Kinase, X-Linked*
*PRKY*	*Protein Kinase, Y-Linked*
*PRORY*	*Proline Rich, Y-Linked*
*PRY*	*PTPN13-like, Y-Linked*
*RBMY*	*RNA binding motif protein, Y chromosome*
*RPS4Y1*	*Ribosomal Protein S4, Y-Linked 1*
*SLY*	*Sycp3-like Y-Linked*
*SMCY*	*Selected Mouse CDNA On Y, Human Homologue of*
*SOX3*	*SRY-Box 3*
*SRY*	*Sex-determining region Y*
*TBL1Y*	*Transducin β Like 1, Y-Linked*
*TGIF2LY*	*TGFB-Induced Factor 2-Like, Y-Linked*
*TMSB4Y*	*Thymosin β 4, Y-Linked*
*TSPY2*	*Testis-specific protein, Y-Linked 2*
*TTTY14*	*Testis-specific Transcript, Y-Linked 14*
*TXLNGY*	*Taxilin γ Pseudogene, Y-Linked*
*USP9Y*	*Ubiquitin Specific Peptidase 9, Y-Linked*
*UTX*	*Ubiquitously Transcribed Tetratricopeptide Repeat Containing, X-Linked*
*UTY*	*Ubiquitously Transcribed Tetratricopeptide Repeat Containing, Y-Linked*
*VCY*	*Variable Charge, Y-Linked*
*ZFY*	*Zinc Finger Protein, Y-Linked*
